# Commenting on the effects of surface treated- and non-surface treated TiO_2_ in the Caco-2 cell model

**DOI:** 10.1186/1743-8977-9-42

**Published:** 2012-11-12

**Authors:** James J Faust, Wen Zhang, Brian A Koeneman, Yongsheng Chen, David G Capco

**Affiliations:** 1 School of Life Sciences, Cellular and Molecular Biosciences, Arizona State University, Tempe, AZ, 85287-4501, USA; 2 Department of Civil and Environmental Engineering, New Jersey Institute of Technology, Newark, NJ, 07102, USA; 3 School of Civil and Environmental Engineering, Georgia Institute of Technology, Atlanta, GA, 30332, USA

**Keywords:** Nanoparticles, Titanium dioxide, Microvilli, Brush-border, Caco-2, Transmission electron microscopy, Scanning electron microscopy

## Abstract

In a recent work published in *Particle and Fibre Toxicology* by Fisichella and coworkers investigating surface-modified TiO_2_ nanoparticle exposure in a model human intestinal epithelium (Caco-2), albeit degraded to mimic conditions in the gut and exposure to natural sunlight, purportedly resulted in no toxic effects. The authors (Fisichella et al.) claim to have confirmed the results of a 2010 report by Koeneman et al. However, the study by Koeneman and colleagues revealed significant effects of unmodified TiO_2_ nanoparticles. These contradicting data warrant further investigation into the possible effects of aluminum hydroxide, as these nanoparticles appear to have resulted in an abnormal apical surface in Caco-2 cells.

This is a comment on http://www.particleandfibretoxicology.com/content/pdf/1743-8977-9-18.pdf.

## Correspondence

In their recent study, Fisichella et al. claim that surface-treated titanium dioxide (TiO_2_) nanoparticles (NPs) do not harm an epithelium composed of Caco-2 cells (an *in vitro* model of human intestinal enterocytes), and claim that these NPs are unlikely to enter the body via the oral route [1]. These authors cite our own work [2] and claim to have confirmed our previous study. The study by Koeneman and coworkers showed that application of a mixture of TiO_2_ NPs composed of rutile and anatase elicited significant effects on the cellular epithelium, and further these TiO_2_ NPs were transported across the Caco-2 epithelium.

Many investigators including ourselves have shown that the apical brush-border of these cells exhibit a well-ordered array of microvilli (MV) [2,3]. The scanning electron micrographs of the epithelium shown by Fisichella and colleagues [1] as their Figure 5 B does not have the appearance of being in good health as these MV are greatly distorted and may even be in the process of being absorbed into the surface of the cells, as was shown by Koeneman and colleagues [2] to be an effect of TiO_2_ NP treatment. Microvilli are a cell specialization to increase the surface area of the absorptive cells of the gut, and further their disappearance likely affect nutrient absorption. Some including ourselves would consider a significant reduction in MV, as a result of TiO_2_ exposure, to be detrimental to the intestinal epithelium. The transmission electron microscopy (TEM) results of Fisichella et al. (their Figure 6) also indicate an affect of nanoparticles on the Caco-2 cell epithelium as the cell surface is amorphous. TEM of a normal Caco-2 epithelium exhibit cells with a polarized cytoplasm containing many electron-dense organelles and cytoplasmic granules with a well ordered array of MV at the cellular apex [3,4]. The images shown by Fisichella and coworkers do not resemble these images and show a significant effect of their NP treatment.

The Fisichella et al. study employed surface-treated TiO_2_ NPs modified by aging the surface coating (PDMS) to; 1) mimic conditions in the gut, and 2) to emulate the effects of natural UV exposure. After these separate aging processes the authors claim that an aluminum hydroxide shell surrounding a TiO_2_ core remains intact. The results of Fisichella et al. [1] suggest that the aluminum hydroxide coating affected the MV as there are marked surface defects depicted in their figures ([1], Figure 5 and Figure 6). In addition, others have shown that chlorine at concentrations comparable to municipal water supplies degrades the protective aluminum hydroxide coating [5]. This is relevant because the authors [1] intended to investigate TiO_2_ NPs that are employed as components of sunscreen that are likely to be used as skin protectants while swimming. Although these conflicting data may be a result of the nanomaterials employed, they underscore the need to further examine any potential deleterious effects of TiO_2_ NPs in the Caco-2 cell model.

## Competing interests

The authors declare that they have no competing interests.

## Authors’ contributions

All authors read and approved the final manuscript.
